# Synergistic Benefits of Butyric Acid and Resistant Potato Starch on Growth and Gut Health in Weaned Pigs

**DOI:** 10.1111/jpn.70054

**Published:** 2026-03-03

**Authors:** Kathryn Ruth Connolly, Torres Sweeney, John V. O'Doherty

**Affiliations:** ^1^ School of Agriculture and Food Science University College Dublin Dublin Ireland; ^2^ School of Veterinary Medicine University College Dublin Dublin Ireland

**Keywords:** butyric acid, microbiota, piglets, prebiotic, weaning

## Abstract

Newly‐weaned piglets face challenges such as reduced feed intake, impaired gut function, and susceptibility to post‐weaning diarrhoea, increasing the need for dietary strategies that support gut health and growth. This study investigated the effects of dietary supplementation with butyric acid (BA), resistant potato starch (PS), and their combination on faecal markers of intestinal health and growth performance in weaned piglets. It was hypothesised that BA and PS would exert beneficial effects, and that their co‐supplementation would yield complementary effects on gut function and growth. In a 2 × 2 factorial design, 96 piglets (28 days of age) were assigned to one of four treatments: (1) control, (2) control +15 g/kg BA, (3) control + 10 g/kg PS, or (4) control +15 g/kg BA and 10 g/kg PS (combination). Growth performance was assessed over 35 days, and faecal samples were collected on Day 8 post‐weaning for microbiota and volatile fatty acid profiling. All supplemented diets improved faecal scores compared to the control (*p* < 0.05), with the combination diet producing the highest average daily feed intake (ADFI), average daily gain (ADG), and final body weight (BW) among treatments, significantly higher than the BA‐only and PS‐only diets (*p* < 0.05), and numerically superior over the control (*p* < 0.10). Moreover, the combination diet had the highest faecal *Faecalibacterium* abundance of all treatments, significantly higher than the BA‐only diet (*p* < 0.05) and numerically superior to the control and PS‐only diet (*p* < 0.10). The BA‐only diet increased faecal *Ruminococcaceae* and reduced *Enterobacteriaceae* abundance, but reduced ADG and BW (*p* < 0.05), and tended to reduce ADFI (*p* < 0.10). The PS‐only diet increased faecal *Prevotella* and butyrate levels, and reduced *Proteobacteria* and isobutyrate levels but reduced ADFI (*p* < 0.05). While BA and PS individually improved faecal microbial and metabolic markers without enhancing growth, their combined supplementation improved both gut health and growth, suggesting a synergistic interaction and a promising nutritional strategy to mitigate the negative effects of weaning.

**Abbreviations**
ADFI: average daily feed intake
ADG: average daily gain
DE: digestible energy
DM: dry matter
FS: faecal scores
GE: gross energy
G:F: gain to feed ratio
OTU: operational taxonomic units
VFA: volatile fatty acid

## 1 Introduction

The gut microbiome plays a critical role in supporting nutritional, physiological, and immunological functions in pigs (Fouhse et al. 2016). Butyrate, a short‐chain fatty acid (SCFA) produced by beneficial gut bacteria through dietary fibre fermentation is particularly important. It serves as the primary energy source for colonocytes and contributes to gut barrier integrity and regulated immune responses (Fu et al. 2019; Plöger et al. 2012; Salvi and Cowles 2021; Zhang et al. 2021). In addition, butyrate helps sustain an anaerobic gut environment that promotes the growth of benefical bacteria and reduces the risk of diarrhoea (Hamer et al. 2007; Jha et al. 2019). However, commercial European weaning typically occurs at 28 days of age, prior to the complete maturation of the swine intestinal microbiome and immune system, leading to a reduction in butyrate‐producing bacteria, lower intestinal buyrate levels and increased inflammation post‐weaning (PW) (Dou et al. 2017; Fouhse et al. 2016; Li et al. 2018; Metallo et al. 2025; Nowland et al. 2021; Zhong et al. 2019). Thus, considering butyrate's vital roles and its decline PW, strategies that enhance intestinal butyrate levels may help alleviate the adverse effects of weaning, improving growth performance and gut health in the absence of in‐feed antimicrobials.

Intestinal butyrate levels have been increased via direct supplementation with exogenous butyrate such as butyric acid (BA) (Connolly et al. 2025c; Guilloteau et al. 2010; Maiuolo et al. 2024), and may also be enhanced indirectly through other dietary strategies, including prebiotics, β‐glucans, organic acids and lactose supplementation (Connolly et al. 2024, Connolly et al. 2025b; Conway et al. 2022; Kiernan et al. 2023; Pierce et al. 2007). Resistant potato starch (PS) in particular, has shown promise as a prebiotic in weaned piglets. It provides an ideal fermentable substrate for benefical microbes, promoting their proliferation and enhancing butyrate production and absorption (Tan et al. 2021; Trachsel et al. 2019), while also reducing the incidence of post‐weaning diarrhoea (PWD) (Heo et al. 2014).

Although exogenous butyrate may offer immediate benefits, its effectiveness depends on continuous dietary inclusion. In contrast, stimulating endogenous butyrate production through prebiotics may lead to more gradual but sustained improvement in intestinal health via alterations in the gastrointestinal microbial community. Despite the demonstrated benefits of each approach independently, no studies to date have investigated their combined effects during the PW period. The combination of exogenous butyrate with prebiotics presents a unique opportunity to enhance intestinal butyrate levels PW. This strategy could mitigate the adverse effects of abrupt commercial weaning while priming the gut for long‐term butyrate production.

Therefore, the objective of this study was to evaluate the effects of dietary supplementation with exogenous butyrate (BA), the butyrate‐stimulating prebiotic resistant potato starch (PS), or their combination, on faecal gut health markers and growth performance in post‐weaned pigs. The hypothesis of this study was both BA and PS individually would enhance gut health and growth performance, and that their combined supplementation would have the greatest positive impact on gut health and growth performance.

## 2 Materials and Methods

The procedures outlined in this study were approved by the University College Dublin Animal Research Ethics Committee (AREC‐20‐22‐ODoherty) and conducted in compliance with Irish legislation (SI no. 543/2012) and the EU Directive 2010/63/EU on animal experimentation.

### 2.1 Experimental Design and Dietary Treatments

The experiment was conducted as a 2 × 2 factorial design using 96 piglets, which were offspring of a Meatline Hermitage boar (Sion Road, Kilkenny, Ireland) and Large White × Landrace sows. The piglets were selected from a commercial swine unit on the day of weaning (28 days old), with an average weaning weight of 7.8 ± 0.72 kg (standard deviation). They were blocked based on weaning weight, litter of origin and sex, and within each block, were randomly assigned to one of four dietary treatments for a 35‐day PW experimental period. The dietary treatments were as follows: (T1) control diet; (T2) (BA) control diet with 15 g/kg encapsulated BA; (T3) (PS) control diet with 10 g/kg PS; and (T4) (combination) control diet with 15 g/kg encapsulated BA and 10 g/kg PS. The encapsulated BA, ButiPEARL (Kemin Industries, Des Moines, IA, USA), was included at a rate of 15 g/kg based on the findings of previous research (Connolly et al. 2025c), while the PS was sourced from Pure Ingredients (Dublin, Ireland) and included at a rate of 10 g/kg (Heo et al. 2014). The diets were formulated to provide similar net energy values (10.6 MJ/kg) and standardised ileal digestible lysine concentration (12.0 g/kg). The amino acid levels were adjusted to meet or surpass the recommendations outlined by NRC (2012). All diets were milled on‐site and offered to the piglets in mash form. The ingredient composition and analysis is presented in Table 1. On the 8th day PW, fresh faecal samples were collected from each pen (*n* = 8) for microbial and volatile fatty acid (VFA) analysis and immediately frozen at –80°C.

**Table 1:** The ingredient composition and chemical analysis of dietary treatments (g/kg).

	Treatments^a^			
Grain preservation method	Control	BA	PS	Combination
Ingredients (g/kg)				
Wheat	340.0	325.0	330.0	315.0
Full fat soya	170.0	170.0	170.0	170.0
Flaked wheat	130.0	130.0	130.0	130.0
Soyabean meal	105.0	105.0	105.0	105.0
Flaked maize	70.0	70.0	70.0	70.0
Whey powder	50.0	50.0	50.0	50.0
Soya oil	65.0	65.0	65.0	65.0
Vitamins and minerals	2.5	2.5	2.5	2.5
Sodium bicarbonate	2.0	2.0	2.0	2.0
Mono calcium phosphate	4.0	4.0	4.0	4.0
Calcium carbonate (Limestone)	6.0	6.0	6.0	6.0
Salt	2.0	2.0	2.0	2.0
Lysine HCL	4.0	4.0	4.0	4.0
DL‐methionine	1.5	1.5	1.5	1.5
l‐threonine	1.5	1.5	1.5	1.5
Butyric acid	—	15	—	15
Resistant potato starch	—	—	10	10
Chemical analysis (g/kg)				
DM	910.93	914.21	909.91	912.78
Ash	52.64	52.69	52.87	53.51
GE (MJ/kg)	16.76	16.77	16.47	17.08
Crude protein	193.50	192.80	192.90	189.50
Crude Fibre	27.33	26.70	26.80	26.50
NDF	63.52	64.04	65.45	64.22
ADF	35.02	34.50	34.60	34.50
Starch	329.05	320.02	333.04	330.01
Ether Extract	107.51	107.03	107.34	107.14
Lysine	13.50	13.30	13.60	13.50
Methionine and cysteine	7.40	7.50	7.20	7.60
Threonine	7.90	7.70	7.10	7.80
Tryptophan	2.60	2.80	2.50	2.80
Calcium	7.10	7.20	7.10	7.30
Phosphorous	6.20	6.00	6.00	6.20

*Note:* Treatments: (control) control diet; (BA) control diet + 15 g/kg of butyric acid; (PS) control diet + 10 g/kg of resistant potato starch; (combination) control diet + 15 g/kg of butyric acid and 10 g/kg of resistant potato starch.

Abbreviations: ADF, acid detergent fibre; DM, dry matter; GE, gross energy; NDF, neutral detergent fibre.

^a^ Vitamin and mineral concentrate (per kg diet): 250 mg choline chloride; 140 mg Fe; 120 mg Zn; 47 mg Mn; 25 mg Cu; 0.6 mg I; 0.3 mg S; 12 mg nicotinic acid; 10 mg pantothenic acid; 67 mg tocopherol; 4 mg menaquinone; 2 mg riboflavin; 2 mg thiamine; 1.8 mg retinol; 0.025 mg cholecalciferol; 0.015 mg pyridoxine; 0.01 mg cyanocobalamin; 500 mg phytase.

### 2.2 Housing and Animal Management

The piglets were housed in groups of three, with eight replicates per treatment in fully slatted pens measuring 16.8 × 1.22 m. The temperature in the weaner houses was regulated at 30°C for the first 7 days and then lowered by 2°C per week for the remainder of the experimental period. The humidity within the houses was maintained at 65% during the experiment. Feed in mash form and water were available *ad libitum* from two‐space feeders and nipple drinkers. The piglets were weighted at the start of the experiment (Day 0) and then every 7 days for the entire study to calculate average daily gain (ADG). Offered feed was also weighted back weekly to calculate average daily feed intake (ADFI), and gain to feed ratio (G:F).

### 2.3 Faecal Scoring

The piglets were monitored carefully for the clinical signs of diarrhoea, and a faecal scoring system was implemented to determine both the occurrence and severity of the diarrhoea, which was previously described by Connolly et al. (2025a). Faecal scoring was carried out twice a day by the same operator using a scale of 1–5. Using this scale: 1 = *hard, firm faeces*; 2 = *slightly soft faeces*; 3 = *soft, partially formed faeces*; 4 = *loose, semi‐ liquid faeces* and 5 = *watery, mucous‐like faeces*.

### 2.4 Feed Analysis

Feed samples were ground using a 1 mm screen (Christy and Norris Hammer Mill, Chelmsford, UK) and then dried at 55°C for 72 h to determine their dry matter (DM) content. The dried samples were weighed and combusted in a muffle furnace (Nabertherm) at 550°C for 6 h to measure crude ash content. Gross energy (GE) were analysed using an adiabatic bomb calorimeter (Parr Instruments, St. Moline, IL, USA). The nitrogen content was assessed using the Leco FP 528 instrument (Leco Instruments, Stockport, UK Ltd). Dietary concentrations of amino acids was determined by HPLC, as detailed by Iwaki et al. (1987). The crude fibre content was determined in accordance with AOAC International (2005; method number 962.09), while neutral detergent fibre (NDF) and acid detergent fibre (ADF) determined utilising the Ankom 220 Fibre Analyser (Ankom Technology, USA) as detailed by Mertens et al. (2002). Dietary starch concentrations were determined using the Megazyme total starch assay procedure (method number 996.11; Association of Official Analytical Chemists; Megazyme, Bray, Co. Wicklow Ireland). The dietary crude fat levels were determined using light petroleum ether and Soxtec instrumentation (Tecator, Hillerod, Sweden). The ingredients and chemical analysis of the diets are presented in Table 1.

### 2.5 Microbiological Analysis

#### 2.5.1 Microbial DNA Extraction

Genomic DNA were isolated from samples using the QIAamp PowerFecal Pro DNA kit (Qiage, West Sussex, UK) following the manufacturer's protocol. The concentration and purity of the extracted DNA was assessed using a Nanodrop ND‐1000 Spectrophotometer (Thermo Scientific, Wilmington, DE, USA).

#### 2.5.2 Illumina Sequencing

High‐throughput sequencing of the V3‐V5 hypervariable region of the bacterial 16S rRNA gene was performed using the Illumina MiSeq platform in accordance with the Eurofins Genomics (Ebersberg, Germany) protocol. Universal primers with adapter overhang sequences were used to PCR‐amplify the V3‐V5 region. AMPure XP beads (Beckman Coulter, Indianapolis, IN) were utilised to purify the amplicons before being set up for Index PCR, which was performed using Nextera XT index primers (Illumina, San Diego, CA). The indexed samples were further purified with AMPure XP beads and then quantified using a fragment analyser (Agilent, Santa Clara, CA). Equal quantities of each sample were pooled and quantified with a Bioanlyser 7500 DNA kit (Agilent, Santa Clara, CA) and sequenced using the v3 chemistry (2 × 300 bp paired end reads).

#### 2.5.3 Bioinformatics

The bioinformatic analysis of the sequences was performed by Eurofins Genomics (Ebersberg, Germany) using the open‐source software package Quantitative Insights into Microbial Ecology (QIIME, Version 1.9.1). Raw reads which passed the standard Illumina chastity filter were demultiplexed on the basis of their index sequences, with a read score >30. The primer sequences were cut at the start of the raw forward and reverse reads. If the primer sequences did not perfectly match, they were eliminated in order to ensure only high‐quality reads. The paired‐end reads were then merged to form a singular long read that covers the entire target region using the software Flash 2.200 (Magoč and Salzberg 2011). The pairs were merged with a minimum overlap of 10 bp to minimise false‐positive merges. If merging was not possible, the forward reads were retained for further analysis. Merged reads underwent quality filtering based on expected and known variations in the V3‐V5 region. Retained forward reads were trimmed to a final length of 300 bp to remove low‐quality bases. Both merged and retained forward reads that contained ambiguous bases were removed. The resulting filtered reads were used for the microbiome profile. Chimeric sequences were detected and eliminated using UCHIME's de novo algorithm (Edgar et al. 2011), which is integrated within the VSEARCH package (Rognes et al. 2016). The remaining high‐quality reads were processes utilising minimum entropy decomposition (MED) to sort them into operational taxonomic units (OTU) (Eren et al. 2013). Taxonomic classification of each OTU was performed by aligning cluster representative sequences to the NCBI database using DC‐MEGABLAST. A sequence identity of 70% over at least 80% of the representative sequence was the requirement in order to be considered a reference sequence. The taxonomic sequences were normalised using linear‐specific copy numbers of the suitable marker gene to enhance estimates (Angly et al. 2014). The complete data matrix, which included the normalised metadata and phylogenetic tree was imported into the R package phyloseq (Version 3.5.0). Differential abundance analysis was then performed at the phylum, family and genus levels using the tables generated by phyloseq.

### 2.6 Volatile Fatty Acid Analysis

Faecal VFA concentrations were assessed with gas‐liquid chromatography as previously detailed by Clarke et al. (2018). One gram of each faecal sample was diluted with distilled water (2.5 times the weight of the sample) and then centrifuged at 1400 x g for 10 min (Sorvall GLC‐2 B laboratory centrifuge; DuPont). After centrifugation, 1 ml of the resulting supernatant was mixed with 1 ml of an internal standard (0.05% 3‐methly‐n‐valerix acid in 0.15 M oxalic acid dihydrate and 3 mL of distilled water). The mixture was centrifuged at 500 g for 10 min before being filtered through a 0.45 polytetrafluoroethylene syringe filter into a chromatographic sample vial. During the gas‐chromatography analysis, 1 μL of the sample was injected into a Varian 3800 GC equipped with an EC 1000 Grace column (15 m × 0.53 mm I.D) with 1.20 μm film thickness. The temperature profile was set as follows: starting at 75°C, the temperature was increased to 90°C at rate of 3°C per minute, then further raised to 200°C at a faster rate of 20°C per minute and held for 0.5 min. The injector temperature was maintained at 240°C, while the detector was set to 280°C. Each sample took 12.42 min to be fully analysed.

### 2.7 Statistical Analysis

The growth performance, faecal score and VFA data were all checked for normality using the Shapiro‐Wilk test in UNIVARIATE of SAS software version 9.4 (SAS Institute, Inc.). If necessary, the data was transformed. The growth performance parameters (ADFI, ADG, G:F and BW) were analysed using PROC GLM of SAS (Software version 9.4, SAS Institute, Inc., Cary, NC, USA). The analysis was conducted for periods: Days 0–21, 21–35 and for the overall experimental period. These periods were selected due to the piglets being the most susceptible to weaning‐related disturbances during the first 21 days PW, and to assess the effects of the dietary treatments across the entire PW period. The statistical model included BA supplementation, PS supplementation and their associated two way interactions. Initial body weight was used as a covariate. The faecal score (FS) data was averaged every 3 days and analysed using the repeated measure analysis of PROC MIXED. The statistical model included BA supplementation, PS supplementation, time and their associated two and three way interactions. The VFA data was analysed using PROC GLM. The statistical model included BA supplementation, PS supplementation and their associated two way interactions. The faecal microbiome data was analysed using PROC GLIMMIX for non‐parametric data, with the results presented as least‐square means, using Benjamini‐Hochberg (BH) adjusted *P*‐values. The experimental unit for all parameters was the pen. The results are presented as least‐square means with their standard errors. The probability level that indicates significance is *p* < 0.05, and a numerical tendency is 0.05 ≤ *p* < 0.10.

## 3 Results

### 3.1 Growth Performance and Faecal Scores

The effect of dietary treatment on body weight (BW), ADG, ADFI, G:F and FS during the PW period (Days 0–21, Days 21–35 and Days 0–35) are presented in Table 2.

**Table 2:** The effect of dietary treatment on growth performance and faecal scores (Least‐square means with their standard errors).

	Treatment^a^					*p*‐value		
	Control	BA	PS	Combination	SEM	BA	PS	BA × PS
**Days 0–21**								
ADFI (g/day)	526^a^	507^a^	508^a^	585^b^	15.64	0.051	0.048	**0.002**
ADG (g/day)	402^bc^	344^a^	365^ab^	416^c^	18.82	0.860	0.319	**0.003**
G:F (g/kg)	769	690	719	714	35.32	0.215	0.702	0.279
BW (kg)	16.28^bc^	15.07^a^	15.52^ab^	16.59^c^	0.396	0.862	0.320	**0.003**
FS	2.50 ^d^	2.35^c^	2.17^a^	2.27^b^	0.031	0.516	< 0.001	**< 0.001**
**Days 21–35**								
ADFI (g/day)	1219^bc^	1116^ab^	1038^a^	1263^c^	42.73	0.136	0.680	**< 0.001**
ADG (g/day)	740	670	671	754	31.37	0.831	0.792	**0.012**
G:F (g/kg)	622	602	657	610	28.66	0.219	0.444	0.612
BW (kg)	26.64^bc^	24.45^a^	24.91^ab^	27.15^c^	0.721	0.974	0.479	**0.002**
**Days 0–35**								
ADFI (g/day)	804^bc^	751^ab^	720^a^	857^c^	23.22	0.060	0.617	**< 0.001**
ADG (g/day)	537^bc^	474^a^	488^ab^	552^c^	20.59	0.969	0.481	**0.002**
G:F (g/kg)	710	655	695	672	27.93	0.148	0.978	0.527

*Note:* Treatments: (control) control diet; (BA) control diet + 15 g/kg of butyric acid; (PS) control diet+ 10 g/kg of resistant potato starch; (combination) control diet + 15 g/kg of butyric acid and 10 g/kg of resistant potato starch. a,b,c,d Mean values within a row with unlike superscript letters were significantly different (*p* < 0.05). Bold values indicate statistically significant.

Abbreviations: ADG, average daily gain; ADFI, average daily feed intake; FS, faecal scores; G:F, gain to feed ratio.

^a^ A total of eight replicates were used per treatment group (replicate = pen, 3 pigs/pen).

During Days 0–21, there were interactions between BA and PS supplementation on ADFI, ADG, BW and FS (*p* < 0.05). The combination diet resulted in the highest ADFI, significantly higher than the BA‐only, PS‐only and control diets (*p* < 0.05). The combination diet resulted in the highest ADG and BW, significantly higher than the BA‐only and PS only diets, and numerically higher than the control diet. The BA‐only diet, PS‐only diet and combination diet had all reduced FS compared to the control (*p* < 0.05). There was no BA, PS or BA × PS effect on G:F.

During Days 21–35 there was an interaction between BA and PS supplementation on BW (*p* < 0.05). The combination diet resulted in the highest BW, significantly higher than the BA‐only and PS‐only diets, and numerically higher than the control. There was no BA, PS or BA × PS effect on G:F.

During Days 21–35 and the overall period, there were interactions between BA and PS supplementation on ADFI and ADG (*p* < 0.05); The combination diet had the highest ADFI, significantly higher than the BA‐only and PS‐only diets and numerically higher than the control. The combination diet had the highest ADG, significantly higher than the BA‐only and PS‐only diets, and numerically higher than the control diet. There was no BA, PS or BA × PS effect on G:F.

### 3.2 Volatile Fatty Acids

The effects of dietary treatment on faecal VFA concentrations and molar VFA proportions are presented in Table 3. Supplementing PS increased molar butyrate (0.118 vs. 0.101, SEM 0.0050) and reduced molar isobutyrate (0.024 vs. 0.031, SEM 0.0024) compared to non‐PS supplemented diets (*p* < 0.05). There was a numerical tendency for BA supplementation to increase molar propionate compared to non‐BA supplemented diets (0.237 vs. 0.214, SEM 0.0085; *p* = 0.066). There was a numerical tendency for PS supplementation to increase total VFA concentrations compared to non‐PS supplemented diets (290.21 vs. 229.84, SEM 23.898; *p* = 0.075). There was no effect of BA supplementation on faecal molar butyrate proportions or total VFA concentrations (*p* > 0.05).

**Table 3:** The effect of dietary treatment on the molar proportions and total concentrations of VFA in faeces (Least‐square means with their standard errors).

	Treatment^a^					*p* value		
	Control	BA	PS	Combination	SEM	BA	PS	BA × PS
Molar proportions								
Acetate	0.598	0.589	0.581	0.564	0.0176	0.425	0.195	0.789
Propionate	0.212	0.222	0.217	0.251	0.0128	0.066	0.1401	0.310
Butyrate	0.099	0.102	0.126	0.109	0.0076	0.319	**0.020**	0.172
Valerate	0.019	0.018	0.024	0.016	0.0039	0.194	0.686	0.355
Isobutyrate	0.031	0.030	0.021	0.026	0.0036	0.529	**0.044**	0.444
Isovalerate	0.041	0.040	0.031	0.034	0.0051	0.890	0.091	0.596
BCFA	0.091	0.087	0.077	0.074	0.0100	0.712	0.154	0.953
Total	204.59	255.09	317.74	262.67	36.130	0.945	0.075	0.117

*Note:* Treatments: (control) control diet; (BA) control diet + 15 g/kg of butyric acid; (PS) control diet + 10 g/kg of resistant potato starch; (combination) control diet + 15 g/kg of butyric acid and 10 g/kg of resistant potato starch. Bold values indicate statistically significant.

Abbreviations: BCFA, branched‐chain fatty acids; VFA, volatile fatty acids.

^a^ A total of eight replicates were used per treatment group.

### 3.3 Differential Bacterial Abundance Analysis

Tables 4, 5, 6 present the effect of dietary treatment on the abundance of specific bacteria in the faecal microbiome.

**Table 4:** The effect of dietary treatment the relative abundance of bacterial phyla in the faeces (mean % relative abundance with their standard errors).

	Treatments^a^					*p* value		
Phylum	Control	BA	PS	Combination	SEM	BA	PS	BA × PS
Faeces								
Firmicutes	82.54^b^	76.77^ab^	67.86^a^	78.42^ab^	3.434	0.389	0.044	**0.014**
Bacteroidetes	9.36^a^	14.55^b^	22.68^c^	13.54^ab^	1.684	0.707	< 0.001	**< 0.001**
Actinobacteria	2.41	1.97	1.96	1.21	0.586	0.220	0.212	0.594
Tenericutes	0.73^a^	2.33^b^	1.78^ab^	1.43^ab^	0.540	0.152	0.533	**0.040**
Proteobacteria	2.67	1.27	0.97	0.58	0.617	0.091	**0.019**	0.757

*Note:* Treatments: (control) control diet; (BA) control diet + 15 g/kg of butyric acid; (PS) control diet + 10 g/kg of resistant potato starch; (combination) control diet + 15 g/kg of butyric acid and 10 g/kg of resistant potato starch. a,b Mean values within a row with unlike superscript letters were significantly different (*p* < 0.05). Bold values indicate statistically significant.

^a^ A total of eight replicates were used per treatment group.

**Table 5:** The effect of dietary treatment the relative abundance of selected bacterial family in the faeces (mean % relative abundance with their standard errors).

Family	Treatments^a^					*p* value		
	Control	BA	PS	Combination	SEM	BA	PS	BA × PS
Faeces								
Lactobacillaceae	6.52	6.29	7.44	11.47	1.197	0.145	**0.010**	0.086
Lachnospiraceae	22.86^c^	18.73^bc^	11.82^a^	15.08^b^	1.807	< 0.001	0.806	**0.020**
Erysipelotrichaceae	0.98	0.45	0.50	0.28	0.404	0.218	0.297	0.839
Eubacteriaceae	2.86^b^	1.40^ab^	1.29^a^	2.96^b^	0.639	0.924	0.825	**0.008**
Ruminococcaceae	27.37	30.41	25.77	33.35	2.042	**0.013**	0.816	0.274
Clostridiaceae	3.22	2.74	2.64	3.54	0.678	0.753	0.885	0.284
Propionibacteriaceae	1.16^ab^	2.11^ab^	2.49^b^	0.43^a^	0.557	0.261	0.121	**0.003**
Oscillospiraceae	4.58	5.71	2.90	3.12	0.845	0.434	**0.008**	0.697
Spiroplasmataceae	0.40^a^	1.87^b^	1.87^b^	1.07^ab^	0.484	0.215	0.217	**0.012**
Rikenellaceae	3.26	4.42	2.85	2.83	0.744	0.474	0.166	0.447
Hungateiclostridiaceae	2.53	1.45	0.92	0.79	0.601	0.291	**0.021**	0.550
Muribaculaceae	2.89^a^	7.65^b^	6.79^b^	2.56^a^	0.978	0.997	0.516	**< 0.001**
Veillonellaceae	2.09	0.19	1.28	2.03	0.547	0.055	0.049	**0.005**
Prevotellaceae	3.55	3.01	11.67	8.33	1.208	0.133	**< 0.001**	0.614
Christensenellaceae	2.96	6.39	2.63	2.91	0.894	**0.036**	**0.042**	0.112
Enterobactriaceae	2.72^b^	0.06^a^	0.96^a^	0.60^a^	0.624	0.012	0.431	**0.045**
Selenomonadaceae	0.08	0.15	0.04	0.13	0.139	0.485	0.758	0.810
Methanobacteriaceae	1.68	2.28	0.17	1.25	0.534	0.028	**0.007**	0.099
Acidaminococcaceae	2.16	1.46	2.27	2.04	0.556	0.345	0.464	0.583

*Note:* Treatments: (control) control diet; (BA) control diet + 15 g/kg of butyric acid; (PS) control diet + 10 g/kg of resistant potato starch; (combination) control diet + 15 g/kg of butyric acid and 10 g/kg of resistant potato starch. a,b, c Mean values within a row with unlike superscript letters were significantly different (*p* < 0.05). Bold values indicate statistically significant.

^a^ A total of eight replicates were used per treatment group.

**Table 6:** The effect of dietary treatment the relative abundance of selected bacterial genera in the faeces (mean % relative abundance with their standard errors).

Genus	Treatments^a^					*p* value		
	Control	BA	PS	Combination	SEM	BA	PS	BA × PS
Faeces								
Lactobacillus	6.76	6.29	7.44	9.12	1.141	0.097	0.629	0.320
Catenibacterium	0.48	0.19	0.28	0.09	0.284	0.233	0.452	0.905
Gemmiger	4.77	4.56	5.59	5.82	0.853	0.984	0.216	0.790
Ruminococcus	1.84	2.10	1.04	0.88	0.513	0.945	**0.031**	0.638
Faecalibacterium	11.56^a,b^	8.83^a^	13.38^a,b^	16.70^b^	1.445	0.824	0.001	**0.028**
Butyricicoccus	2.81	2.39	2.45	3.21	0.634	0.806	0.720	0.335
Holdemanella	0.02	0.09	0.22	0.16	0.164	0.731	0.414	0.618
Clostridium	0.22	0.23	0.25	0.33	0.204	0.813	0.732	0.874
Oscillibacter	4.82	5.71	3.04	3.14	0.845	0.586	**0.007**	0.710
Spiroplasma	0.41^a^	1.86^b^	1.88^b^	1.08^ab^	0.485	0.227	0.220	**0.013**
Propionibacterium	1.21^a,b^	2.11^a,b^	2.48^b^	0.43^a^	0.557	0.108	0.239	**0.003**
Anaerocella	2.37	1.53	3.13	1.51	0.625	**0.032**	0.615	0.569
Eubacterium	3.02^b^	2.37^ab^	1.40^a^	2.96^b^	0.656	0.298	0.266	**0.049**
Anaerobacterium	1.04	0.64	0.46	0.48	0.386	0.635	0.249	0.569
Prevotella	3.66	2.98	11.12	7.96	1.179	0.113	**< 0.001**	0.695
Phascolarctobacterium	2.26	1.46	2.24	2.04	0.568	0.314	0.532	0.509
Roseburia	2.07	2.25	2.63	3.14	0.627	0.578	0.223	0.834
Fournierella	1.86^a,b^	2.67^b^	2.33^a,b^	1.04^a^	0.577	0.422	0.220	**0.041**
Megasphaera	1.33^b^	0.19^a^	1.28^b^	2.02^b^	0.502	0.022	0.1309	**0.019**
Agathobacter	2.67^a,b^	1.98^a,b^	1.47^a^	3.04^b^	0.617	0.385	0.732	**0.047**
Blautia	4.44	3.81	3.31	2.57	0.797	0.308	0.088	0.801
Kineothrix	0.44	0.96	0.50	1.41	0.420	0.060	0.580	0.784
Christensenella	2.65	5.81	2.82	2.77	0.853	0.077	0.115	0.066
Pseudoflavonifractor	3.80	6.91	3.53	6.65	0.930	**< 0.001**	0.740	0.923
Coprococcus	5.64	4.07	1.62	0.63	0.898	**0.038**	**< 0.001**	0.299
Methanobrevibacter	1.82	2.28	0.17	1.27	0.534	**0.031**	**0.006**	0.081
Duncaniella	2.06^a^	7.65^b^	6.70^b^	2.55^a^	0.970	0.385	0.838	**< 0.001**
Intestinimonas	0.45	0.60	0.47	0.16	0.273	0.535	0.317	0.291
Anaerobutyricum	0.85	0.79	0.41	0.86	0.348	0.451	0.475	0.361
Oribacterium	0.12	0.33	0.68	1.02	0.358	0.296	**0.047**	0.644
Ruminiclostridium	0.17	0.65	0.33	0.08	0.285	0.956	0.421	0.131
Mediterraneibacter	1.72	0.38	0.09	0.17	0.496	0.576	**0.028**	0.195

*Note:* Treatments: (control) control diet; (BA) control diet + 15 g/kg of butyric acid; (PS) control diet + 10 g/kg of resistant potato starch; (combination) control diet + 15 g/kg of butyric acid and 10 g/kg of resistant potato starch. a, b Mean values within a row with unlike superscript letters were significantly different (*p* < 0.05). Bold values indicate statistically significant.

^a^ A total of eight replicates were used per treatment group.

### 3.4 Differently Abundant Phyla

The predominant faecal phyla in all of the dietary treatments were Firmicutes (∼76.20%), Bacteroidetes (∼15.22%), Actinobacteria (∼2.37%), Proteobacteria (∼1.86%) and Tenericutes (∼1.60%).

There were interactions between BA and PS on the relative abundance of Firmicutes, Bacteroidetes and Tenericutes (*p* < 0.05); Piglets offered the PS diet had reduced relative abundance of Firmicutes compared to the control diet, but there was no difference in Firmicutes between the combination diet and the BA diet on Firmicutes. The BA diet had increased relative abundance of Bacteroidetes compared to the control diet, but the combination diet had reduced Bacteroidetes compared to the PS only diet. Piglets offered the BA diet had increased relative abundance of Tenericutes compared to the control diet, however there was no difference in Tenericutes between the combination diet and the PS only diet. Piglets offered PS had reduced relative abundance of Proteobacteria (0.75 vs. 1.84, SEM 0.359; *p* < 0.05) compared to non‐PS supplemented diets.

### 3.5 Differentially Abundant Families

There were interactions between BA and PS supplementation on *Lachnospiraceae, Eubacteriaceae, Propionibacteriaceae, Spiroplasmataceae, Muribaculaceae, Veillonellaceae* and *Enterobacteriaceae* (*p* < 0.05); The PS only diet had reduced relative abundance of *Lachnospiraceae*, *Eubacteriaceae* and *Enterobacteriaceae* compared to the control diet, but there was no difference in *Lachnospiraceae*, *Eubacteriaceae* and *Enterobacteriaceae* between the combination diet and the BA only diet. The BA diet had increased relative abundance of *Spiropasmataceae* compared to the control diet, but there was no difference in *Spiropasmataceae* between the combination diet and the PS only diet. Piglets offered the combination diet had reduced relative abundance of *Propionibacteriaceae* compared to the PS diet, but there was no difference in *Propionibacteriaceae* between the control and the BA diet.

Piglets offered BA had increased relative abundance of *Rumincoccaceae* (31.85 vs. 26.56, SEM 1.412) and *Christensenellaceae* (4.31 vs. 2.79, SEM 0.599) compared to non‐BA supplemented diets (*p* < 0.05). Piglets offered PS had increased relative abundance of *Lactobacillaceae* (9.24 vs. 6.40, SEM 0.769) and *Prevotellaceae* (9.86 vs. 3.26, SEM 0.791) compared to non‐PS supplemented diets (*p* < 0.05). Piglets offered PS had reduced relative abundance of *Oscillospiraceae* (3.01 vs. 5.11, SEM 0.589), *Hungateiclostridiaceae* (0.85 vs. 1.91, SEM 0.362), *Christensenellaceae* (2.77 vs. 4.35, SEM 0.539) and *Methanobacteriaceae* (0.47 vs. 1.96, SEM 0.366) compared to non‐PS supplemented diets (*p* < 0.05).

### 3.6 Differentially Abundant Genera

There were interactions between BA and PS on the relative abundance of *Faecalibacterium, Spiroplasma, Propionibacterium, Eubacterium, Fournierella, Megasphaera, Agathobacter* and *Duncaniella* (*p* < 0.05); The BA diet had reduced relative abundance of *Faecalibacterium* compared to the control diet, but there was no difference in *Faecalibacterium* between the combination diet and the PS only diet. The BA diet had increased relative abundance of *Spiroplasma* compared to the control diet, but there was no difference in *Spiroplasma* between the combination diet and the PS only diet. The combination diet had reduced relative abundance of *Propionibacterium* and *Fournierella* compared to the PS diet, however there was no difference in *Propionibacterium* and *Fournierella* between the BA only diet and the control diet.

The combination diet had increased relative abundance of *Eubacteirum* and *Agathobacter* compared to the PS diet, but there was no difference in *Eubacterium* and *Agathobacter* between the BA and the control diet. The BA diet had reduced relative abundance of *Megasphaera* compared to the control diet, however there was no difference in *Megasphaera* between the combination diet and the PS only diet. The BA diet had increased relative abundance of *Duncaniella* compared to the control diet, but the combination diet had reduced *Duncaniella* compared to the PS diet.

The supplementation of BA increased the relative abundance of *Pseudoflavonifractor* (6.78 vs. 3.66, SEM 0.605) and *Methanobrevibacter* (1.70 vs. 0.56, SEM 0.372) compared to non‐BA supplemented diets (*p* < 0.05). The supplementation of BA reduced the relative expression of *Anaerocella* (1.52 vs. 2.72, SEM 0.431) and *Coprococcus* (1.60 vs. 3.02, SEM 0.484) compared to non‐BA supplemented diets (*p* < 0.05). The supplementation of PS reduced the relative abundance of *Ruminococcus* (0.95 vs. 1.96, SEM 0.364), *Oscillibacter* (3.09 vs. 5.24, SEM 0.595), *Coprococcus* (1.01 vs. 4.79, SEM 0.589), *Methanobrevibacter* (0.47 vs. 2.04, SEM 0.372) and *Mediterraneibacter* (0.12 vs. 0.81, SEM 0.274) compared to non‐PS supplemented diets (*p* < 0.05). The supplementation of PS increased the relative abundance of *Prevotella* (9.41 vs. 3.30, SEM 0.772) and *Oribacterium* (0.83 vs. 0.20, SEM 0.231) compared to non‐PS supplemented diets (*p* < 0.05).

## 4 Discussion

Weaning can reduce beneficial gut bacteria populations (Gresse et al. 2019; Li et al. 2018), and butyrate production (Zhong et al. 2019), compromising intestinal function, integrity and homeostasis (Hamer et al. 2007). This study evaluated the effects of dietary supplementation with BA, PS or their combination on faecal gut health markers and growth performance in post‐weaned pigs. It was hypothesised that both BA and PS individually would enhance gut health and growth performance, and that their combined supplementation would provide the greatest benefit due to complementary effects. Supplementing BA had no effect on faecal VFA levels but reduced *Enterobacteriaceae* abundance while increasing *Ruminococcaceae*. However, the BA‐only diet significantly reduced ADG and final BW and tended to reduce ADFI. In contrast, PS supplementation increased faecal butyrate and *Prevotella* levels, while reducing isobutryate and Proteobacteria levels. Despite this, the PS‐only diet had significantly reduced ADFI, particularly between Days 21–35. The combination diet maintained the beneficial effects of the individual additives on gut health while achieving the highest ADFI, ADG and final BW among all treatments, significantly higher than the BA‐only and PS‐only diet and numerically superior to the control. Additionally, all supplemented diets had improved FS compared to the control. These results indicate that combining BA and PS preserved their beneficial effects on faecal gut health markers while mitigating their negative impacts on growth performance, suggesting a promising post‐weaning strategy, especially in more challenging commercial settings.

In this study, BA supplementation was expected to increase intestinal butyrate levels, however this effect was not evident in the faecal VFA profile, where butyrate levels remained unchanged. This was unexpected as a similar study using a 3% inclusion of the same BA additive reported elevated colonic butyrate levels in piglets 10 days PW (Connolly et al. 2025c). This underscores a key limitation of utilising faecal SCFA measurements, which are influenced by microbial production, epithelial absorption and intestinal transit time, making their interpretation complex (Bauer et al. 2004). Therefore, the lack of change in faecal butyrate levels reported in this study may be the result of efficient intestinal absorption in the colon rather than a true absence of increased production. Despite the lack of a faecal butyrate response, BA supplementation improved FS, consistent with previous studies using a 0.2% inclusion level (Feng et al. 2018). This improvement may be partly attributed to shifts in the faecal microbiota. Specifically, in this study, BA reduced *Enterobacteriaceae*, a family belonging to the phylum Proteobacteria, commonly associated with mucosal inflammation, toxin production, gut dysbiosis and systemic infection (Litvak et al. 2017; Pieper et al. 2006; Shin et al. 2015). In addition, BA increased the abundance of *Ruminococcaceae*, a SCFA‐producing family, suggesting a shift towards a more fermentative, anaerobic microbial milieu. However, the functional roles of *Ruminococcaceae* are species‐specific and context dependent, with both beneficial and opportunistic members reported (Valentino et al. 2024). Further research is needed to examine the microbial and VFA‐modulating effects of BA at more targeted regions of the gastrointestinal tract such as the ileum, caecum and colon to gain a more comprehensive understanding of its potential benefits.

The improved FS and faecal microbial changes suggest BA supplementation may benefit gut health, however these changes did not translate into improved growth performance. Growth performance effects were similar across the two different time periods and the overall experimental period. Pigs offered the BA‐only diet had reduced overall ADG, and final BW along with a numerical reduction in overall ADFI. While previous research has reported the growth‐promoting effects of 0.8% BA (Piva et al. 2002), others have noted neutral or even negative outcomes when BA was included at lower inclusion levels of 0.1, 0.2% and 0.4% (Biagi et al. 2007). The observed reduction in feed intake may be related to the sour organoleptic profile of BA (Cheon and Mattes 2020), as well as its known appetite‐suppressive action via the stimulation of peptide YY (*PYY*) (Hamer et al. 2007). These findings highlight the need to determine an optimal BA inclusion level that supports gut health without compromising feed intake or growth in weaned pigs.

While the effects of BA supplementation appear to be predominantly anti‐microbial, PS supplementation exhibited a more pronounced prebiotic effect, as evidenced by changes in faecal VFA and microbiome profiles. In this study, supplementing PS elevated molar butyrate concentrations and reduced isobutyrate, an indicator of proteolytic fermentation (Rios‐Covian et al. 2020), reflecting a shift toward saccharolytic fermentation. This shift was accompanied by a higher abundance of *Prevotella* and *Prevotellaceae*, suggesting enhanced carbohydrate fermentation and improved nitrogen utilization (Le Sciellour et al. 2018; Mach et al. 2015). Additionally, PS supplementation reduced Proteobacteria, indicating these changes may have contributed to a healthier gut environment, as supported by the PS‐only diet, achieving the best FS value of all treatments. This outcome aligns with previous findings where PS reduced diarrhoea by promoting a favourable microbial ecosystem (Bhandari et al. 2009; Heo et al. 2014). However, the PS‐only diet also significantly reduced ADFI, particularly during Days 21–35. This is consistent with research reporting that the fermentation of resistant starch elevates SCFA levels and promotes satiety through gut‐brain axis mechanisms (Tan et al. 2019). Nonetheless, PS did not significantly affect ADG or final BW. The reduction in feed intake may have attenuated its potential benefits on growth, suggesting a complex interplay between microbial modulation and energy intake.

This study hypothesised that the combination of BA and PS would yield the most beneficial effects on gut health and growth performance due to their complementary effects. In agreement with this, the combination diet preserved the positive impacts of each additive on faecal VFA profiles, faecal microbial composition and FS while avoiding the growth performance reductions observed with BA or PS alone. Additionally, the combination diet resulted in the highest abundance of faecal *Faecalibacterium* of all treatments. This genus is well known for its anti‐inflammatory activity, tight junction promoting properties and ability to support mucosal immunity (Ferreira‐Halder et al. 2017; He et al. 2021; Massacci et al. 2020). Elevated levels of *Faecalibacterium* may also enhance energy harvesting through cross‐feeding mechanisms, which may help explain the improved ADFI and ADG observed. Supporting this, recent studies have consistently linked increased *Faecalibacterium* with superior nutrient digestibility and growth performance in pigs (Maher et al. 2024, 2025). Notably in this study, the combination diet had the highest ADFI, ADG and final BW of all treatments. These improvements were significant relative to both the individual additives and numerically superior to the control. This suggests that co‐supplementation may have mitigated the appetite‐suppressive effects reported with BA an PS alone, possibly through balanced fermentation kinetics, enhanced gut health or modulation of satiety signalling. Moreover increased feed intake may have improved the effective delivery and utilisation of both additives, amplifying their functional benefits.

Although nutrient digestibility and regional sampling of gut segments for functional assays such as intestinal permeability, cytokine expression, and mucosal histology were not measured in this study, they represents a key avenue for future research to better understand the mechanisms underpinning the observed growth improvements. Additionally, the current study was conducted under high‐health experimental conditions that may not truly reflect the environmental and pathogenic challenges encountered in commercial production systems. It is therefore plausible that the benefits of the combination diet, particularly in supporting growth and mitigating diarrhoea would be even more pronounced under commercial conditions.

## 5 Conclusions

This study demonstrated that dietary co‐supplementation with BA and PS is more effective at improving post‐weaning performance in pigs than either supplement alone. When provided individually, both supplements improved faecal consistency and altered gut microbial activity but failed to support growth and tended to reduced ADFI. In contrast, the combination diet overcame these limitations, yielding the highest ADFI, ADG, and final BW while maintaining improved gut health indicators. These findings support the hypothesis that BA and PS exert complementary effects on the gut microbiota, microbial fermentation, and host physiology. The combined supplementation of BA and PS represents a promising strategy to support post‐weaning growth and intestinal health without antimicrobial growth promoters, with further validation under commercial production conditions required.

## Author Contributions

**Kathryn Ruth Connolly:** conceptualisation, methodology, investigation, data curation, formal analysis, writing – original draft, writing – review and editing. **Torres Sweeney:** resources, writing – review and editing. **John V. O'Doherty:** conceptualisation, methodology, resources, formal analysis, writing – review and editing, funding acquisition, supervision. All authors approved the final manuscript.

## Conflicts of Interest

The authors declare no conflicts of interest.

## Data Availability

The data that support the findings of this study are available from the corresponding author upon reasonable request.
